# Changes in posttraumatic growth, core belief disruption, and social support over the first year of the COVID-19 pandemic

**DOI:** 10.3389/fpsyg.2022.1019273

**Published:** 2022-10-10

**Authors:** Whitney Dominick

**Affiliations:** Psychology Department, Oakland University, Rochester, MI, United States

**Keywords:** COVID-19, post-traumatic growth, social support, coping strategies, pets, core belief disruption

## Abstract

Post-traumatic Growth (PTG) is the positive psychological change that may occur after a highly stressful situation that shakes a person's core beliefs about the world. During 2020, the United States experienced the COVID-19 pandemic and a highly contentious political election, both of which have the potential to disrupt core beliefs and evoke perceptions of PTG. Post-traumatic growth, core belief disruption, perceived social support from humans and pets, coping strategies, and stressful events were assessed in 201 participants from the United States (*M*_*age*_: 35.39, *SD*: 14.60) at four time points from April 2020 (T1) until April 2021 (T4). While total PTG did not significantly change from Time 1 to Time 4, perceptions of personal strength and new possibilities increased, as did core belief disruption, and the use of coping strategies decreased. Higher PTG was reported by those who owned pets, those who knew someone who had been hospitalized due to COVID-19, and those who knew someone who had died of COVID-19. While rating COVID-19 or politics as the most stressful event at Time 4 did not correspond to differences in PTG, those who perceived the event to be resolved had higher PTG than those who perceived the event to be ongoing. Having COVID-19 personally and vaccination status was not associated with differences in Post-traumatic Growth. PTG at Time 4 was predicted by core belief disruption and social support in the full sample and in the pet owners only sample, and by support from video conferencing for the full sample only. Time 4 PTG was also predicted by core belief disruption, problem-focused coping, and avoidance coping. Results are discussed in terms of the PTG theoretical model. Additionally, implications for interventions aimed at fostering psychological growth, including through non-traditional forms of social support (i.e., remote communication and perceived support from pets) are addressed.

## Introduction

The 2019 Novel Coronavirus which caused the COVID-19 pandemic is an unprecedented global health crisis that has warranted research examining its effects on mental health worldwide. The implementation of policies such as social distancing, quarantine, and new hygiene practices have caused many to redefine their daily routines and has contributed to a large disruption in the global economy. Although life for many has returned to a “new normal,” the global death toll has surpassed six million (Our World in Data, 2022) and disruptions to the global economy continue. Over one million of those deaths have occurred in the United States, where COVID-19 is the third leading cause of death behind heart disease and cancer (Shiels et al., 2022). According to a scientific brief released by the World Health Organization (2022), the pandemic has led to over a 25% increase in cases of major depressive disorder and anxiety worldwide, and nearly one-third of adults in the United States have reported so much stress from the pandemic that they struggle to make basic decisions (American Psychological Association, 2021).

Simultaneously, the United States experienced one of the most contentious presidential elections in decades, with more than two-thirds of adults in the U.S., regardless of political orientation, stating that the 2020 presidential election was a significant source of stress in their lives, up from just over half for the 2016 election (Bethune, 2020a). Unfortunately, that stress dissipated for only 17% of adults in the United States following the election, with another 27% indicating that their stress had increased (Bethune, 2020b).

### Post-traumatic growth

While there are clear negative mental health impacts resulting from the COVID-19 pandemic and political environment, it is also possible that some people will experience Post-traumatic Growth (PTG; Tedeschi and Calhoun, 1996), the positive psychological changes resulting from the struggle with a highly stressful life event. PTG is typically experienced as a greater appreciation for life, a stronger sense of spiritual connection or faith, the recognition of new opportunities, an acknowledgment of personal strength, and/or a stronger sense of connection with others. According to the PTG theoretical model, for PTG to occur, a person's assumptive beliefs about the world must be challenged, following which, with the help of social support, reflection and meaning making, and time, one may be able to rebuild their core beliefs and experience growth (Calhoun and Tedeschi, 2006). Research conducted during the pandemic has indicated that frontline nurses working with COVID-19 patients in China experienced PTG (Cui et al., 2021), as did patients in Shanghai who had been diagnosed with COVID-19 (Sun et al., 2021) and pregnant Arab women (Chasson et al., 2022). Additionally, a cross-sectional study conducted in August of 2020 indicated that adults in the United States also reported PTG, and that PTG was positively related to perceptions of social support (Northfield and Johnston, 2022).

### Social support

Many avenues of social support are linked to PTG experiences. Support from family, friends, and significant others is positively correlated with PTG (Bozo et al., 2009). Seeking social support in general was found to mediate the relationship between trauma intensity and PTG in those who experienced the loss of a child (Ogińska-Bulik and Kobylarczyk, 2019) and to mediate between interpersonal trauma and PTG and between the experience of multiple trauma types and PTG (Brooks et al., 2019). While typically social support is assessed as in-person support, it may also be possible to experience social support through virtual communication, especially for those who are more isolated. To that end, video conferencing was shown to provide emotional support for frontline workers during the start of the pandemic (Viswanathan et al., 2020), to increase aspects of social support in long-term care residents (Siniscarco et al., 2017), and to improve wellbeing in isolated elderly in Finland (Airola et al., 2020). However, technical issues and lack of familiarity with technology does reduce the benefits of video communication (Siniscarco et al., 2017; Airola et al., 2020). Moreover, video communication precludes physical touch, a component that may play a key role in perceptions of social support. Indeed, physical touch has been found to increase perceptions of empathy (Montague et al., 2013), reduce anxiety (Gagne and Toye, 1994), and buffer against stress (Coan et al., 2006). Previous research on quarantines, which limit physical touch between humans, has found increased anxiety, anger, and post-traumatic stress symptoms because of the quarantine (Brooks et al., 2020).

Despite limited human contact during the COVID-19 pandemic, pets, and physical contact with pets, remained stable and may also contribute to a sense of social support. Owning pets can increase both perceived and actual social support, as well as facilitating social capital (e.g., connectivity between people), indicating that those with pets may have larger support networks than those who are not pet owners (Wood et al., 2014). Indeed, a systemic review of the relationship between pet ownership, loneliness, and social isolation found that owning a pet was associated with lower levels of social isolation and that after the outbreak of COVID-19 pet ownership contributed to lower levels of loneliness (Kretzler et al., 2022). While pet ownership alone may provide benefits, the degree of attachment to pets may also contribute to a sense of social support. Pet attachment mediated the relationship between general health and loneliness in older women (Krause-Parello, 2008), was positively correlated with life satisfaction in adults over 40 (Fu and Zheng, 2009), and corresponded with a higher sense of emotional support in adults from India (Joseph et al., 2019). Additionally, adolescents who spent more time with pets, which has been correlated to higher pet attachment (Joseph et al., 2019), reported higher PTG in the domain regarding social connections (Dominick et al., 2019). Chinese adults who spent more time caring for a pet also displayed increased attachment to that pet, which in turn reduced stress in the owners (Wu et al., 2018).

### Coping strategies

Beyond seeking social support, the upheaval of 2020 may also have prompted the use of various other coping strategies, some of which have been linked to the experience of PTG. In breast cancer survivors, the use of positive coping skills was linked to the experience of PTG both 6 months and 2 years after treatment ended, although the link disappeared 7 years after treatment (Hamama-Raz et al., 2019). This same study found that participants who expressed higher levels of PTG subsequently engaged in more positive coping strategies, and that negative coping was unrelated to the experience of PTG. A meta-analysis examining coping strategies and PTG found that positive reappraisal coping had the largest impact on increasing PTG, with social support coping moderately related to PTG and acceptance coping yielding the smallest, yet still significant, impact on PTG (Prati and Pietrantoni, 2008). However, an examination of mediation models found that lower avoidance coping after experiencing multiple types of traumas or after experiencing childhood trauma was associated with higher PTG (Brooks et al., 2019), and in Greek healthcare workers and 911 telecommunicators, adaptive coping strategies, such as emotional-focused coping, and maladaptive coping strategies, such as avoidant coping, both contributed to PTG (London et al., 2017; Kalaitzaki and Rovithis, 2021). Given the conflicting impacts of different coping strategies, additional examination of their impact on PTG is called for.

### Current study

The current study examines the impact of several types of social support and of coping strategies on the experience of PTG from April 2020 through April 2021. Traditional human support, support through video conferencing, support perceived through pets, and the impact of physical touch with pets are evaluated. The relative impact of problem-focused coping, emotion-focused coping, and avoidant coping on PTG are also assessed. Additionally, due to the chaotic nature of the year, differences in PTG between those most impacted by the pandemic and those most impacted by politics are examined. This study adds valuable insights into the importance of alternative methods of gaining social support and on the impact of both the nature of the stressor and coping strategies utilized, as well as overall ability to recognize positive mental growth following a highly stressful year in the United States.

## Method

### Participants

There were 201 participants who completed four surveys over the course of the first year of the pandemic. Participants ranged in age from 18 through 81, with a mean age of 35.39 (*SD* = 14.60). Participants who did not complete all four time points (*n* = 797) were excluded from analyses. See Table 1 for complete demographics.

**Table 1:** Participant demographics.

**Variable**	**Participants (*N* = 201)**	**Variable**	**Participants (*N* = 201)**
Age	35.39 (14.56)	Essential worker	35.3% Essential workers 31.5% Live with essential worker
Sex	77.6% Female	High risk	28.9% High risk 33.9% Live with high risk individual
Pets	71.0% Own pets	Living status	17.4% Live alone 54.7% Live with romantic partner 13.9% Live with parents 13.9% Live with roommates
Race	83.6% White 4.5% Mixed 4.0% Asian 2.5% Latinx/Hispanic 2.0% Middle Eastern 1.5% Black	Relationship status	37.7% Married 31.2% Dating/in relationship 26.6% Single 3.0% Divorced
State	25.4% Michigan 12.4% Colorado 9.5% California 4.5% Virginia 4.0% Texas 4.0% Utah 4.0% New York	Employment	75.2% Employed 8.0% Unemployed/unable to work 6.0% Students 6.0% Retired 2.5% Out of work & looking for work 2.5% Employed but not working
Religion	47.5% Agnostic/Atheist 36.5% Christianity 6% Unsure 2.5% Judaism 2.0% Buddhism	Had COVID	11.6% Yes
Vaccination status	92.9% Vaccinated or plan to get vaccinated 6.5% Not vaccinated & no plan for vaccination	Knew someone hospitalized	54.8% Yes
		Knew someone who died	42.7% Yes

### Procedure

Participants were recruited through a midwestern university's undergraduate subject pool as well as through snowball sampling that was advertised on social media sites such as Instagram and Reddit. A total of 1,000 participants were issued the T1 online survey starting March 31st, 2020, and were sent follow-up surveys on April 30th, 2020 (T2), September 30th, 2020 (T3), and March 31st, 2021 (T4). Participants who enrolled through the subject pool earned research credit for completing the initial survey. To encourage retention of participants each participant was sent up to three reminder emails every seven days after the initial follow-up survey invitation. In addition, participants were entered into raffles for a $50, $75, and $150 e-gift card for the T2, T3, and T4 surveys, respectively. Demographics and COVID-19 exposure were assessed first. If participants were pet owners, they were asked about the social support provided by their pets. Questionnaires regarding human social support, PTG, and core-beliefs were presented in a randomized order following the demographic and pet support sections. Subsequently, T2, T3, and T4 targeted changes in participants' responses between surveys. All the questionnaires that were included in T1 were also included in the subsequent surveys, with modifications made to the instructions to reflect changes in timing. Questions assessing the use of video support were added to the T2 and ensuing surveys. Because the United States continued to experience disruptions over the summer and fall of 2020, including a racial justice movement and contentious presidential election, questions assessing experiences with several types of stressful events and their relative importance were added to the T3 and T4 surveys. Vaccinations for COVID-19 became available to the public in early 2021, so questions regarding vaccination status were also added to the T4 survey. Ethical approval for this study was granted by the university's internal review board.

### Measures

#### Social support: Pets

Pet ownership was first assessed by asking participants to indicate whether they owned a pet. If they did, the 23-item Lexington Pet Attachment Scale (α = 0.894; Johnson et al., 1992) was used to assess pet attachment as a proxy for social support. Items were rated on a scale from 1 (*strongly agree*) to 4 (*strongly disagree*), with lower scores indicating higher attachment levels. It included items such as “Quite often I confide in my pet.” Four additional questions regarding the use of pets for social support specifically were included. These items ranged from 1(never) to 5 (always), and included items such as “How often have you considered your pet a source of social support in the past week?” These questions had adequate reliability, α = 0.858. The use of touch with pets for social support was assessed by two items (α = 0.801) ranging from 0 (never) to 4 (always), and included items such as “How often has physically touching your pet provided comfort to you in the past week?”

#### Social support: Humans

The Multidimensional Scale of Perceived Social Support (Cheng and Chan, 2004) was used to measure human social support during the COVID-19 pandemic. On a 7-point scale ranging from 1 (*very strongly disagree*) to 4 (*very strongly agree*), participants indicated the human support they had from special people, family, and friends (e.g., “I get the emotional help and support I need from my family”). Scores were averaged for an overall score of human social support, and displayed high reliability, α = 0.929. The use of video conferencing tools for social support was assessed by 8 items ranging from 0 (not at all) to 3 (a lot), included items such as “Has video conferencing allowed you to feel connected with others?” and displayed adequate reliability (α = 0.778). One additional item assessed the average frequency of video conferencing use per week.

#### Coping strategies

Coping strategies were assessed with the brief version of the COPE scale (Carver, 1997). This measure consists of 28 items (α = 0.801) rated on a scale from 1 (not at all) to 4 (a lot) and included items such as “I've been taking action to try to make the situation better.” A separate score was calculated for each of the three subscales: avoidant coping (α = 0.685), emotion-focused coping (α = 0.548), and problem-focused coping (α = 0.816).

#### Stressful events

Participants were asked to assess experiences of, and stress caused by nine events on the T3 and T4 surveys using a sliding scale ranging from −1 to 10. The scale ranged from 0 (no stress, this has not impacted my life) to 10 (extreme stress, this has drastically impacted my life), with −1 indicating no experience with the event. Events included COVID-19, racial injustice, environmental concerns, politics/the November election, death of a loved one, illness/injury, romantic relationship breakup, and other events not listed above. In addition to rating stress from each event, participants were asked to select the event that impacted their life the most or caused them the most stress.

#### Post-traumatic growth

An expanded version of the PTG Inventory (PTGI-X; Tedeschi et al., 2017) consisted of 25-items that measure the degree to which the participants have experienced personal growth as a result of the COVID-19 pandemic or most stressful experience during the time of the study (e.g., “I changed my priorities about what is important in life”). The participants used a 6-point scale ranging from 0 (did not experience this change) to 5 (very great degree). Scores were averaged for a total PTG score (α = 0.956), as well as a mean PTG score for each of the five domains (e.g., Relating to Others; α ranged from 0.844 through 0.889).

#### Challenged core beliefs

The Core Beliefs Inventory (CBI; Cann et al., 2010) consisted of 9 items used to measure the degree to which the COVID-19 pandemic had caused participants to seriously examine their beliefs (e.g., “Because of COVID-19, I seriously examined the degree to which I believe things that happen to people are fair”). Participants rated the items on a 6-point scale ranging from 0 (not at all) to 5 (very great degree). Scores were averaged for a total core-belief disruption score and showed good reliability, α = 0.886.

### Data analysis

Data was analyzed using SPSS 26. Preliminary correlations between T4 variables were assessed using Pearson's Correlation coefficient. Experiences with COVID-19, comparisons based on demographic information, and comparisons between those who completed all four time points and those who completed three or less time points were assessed using Independent Sample *T*-tests. Changes over time were assessed with Paired Sample *t*-tests and repeated measure ANOVAs using a repeated contrast. The Greenhouse-Geisser correction was used if Mauchly's test indicated a violation of the sphericity assumption for the repeated measure ANOVAs. Three hierarchical regressions were used to assess the relative impact of different methods of social support and different coping strategies on Time 4 Post-traumatic Growth. The first regression assessed the impact of social support for all participants, the second assessed the impact of social support for pet owners only, and the third assessed the impact of coping strategies on PTG for the full sample. For the social support regressions, age, sex, household count, and core belief disruption were entered in the first step, pet ownership (for the full sample) or pet attachment, support from pets, and touch with pets (for the pet owners only sample) were entered in the second step, and human social support, support from video conferencing and frequency of video conferencing were added in the third step. For the coping regression, age, sex, and household count were entered in the first step, core belief disruption was entered in the second step, and all three coping strategies were entered in the third step.

## Results

Total PTG at Time 4 was correlated with core belief disruption, all forms of social support, and with both problem-focused and emotion-focused coping strategies. Core belief disruption was correlated with attachment to pets and all three coping strategies. Support from pets was weakly correlated with support from humans and video support, and video conferencing support was correlated with problem-focused and emotion-focused coping. All three coping strategies were moderately correlated with each other. Please see Table 2 for a complete correlation matrix and mean scores of the Time 4 variables.

**Table 2:** Correlations and mean scores.

	**PTG** **(0–5)**	**CBI** **(1–6)**	**Pet attach** **(1–4)**	**Pet support** **(1–5)**	**Social support** **(1–7)**	**Video support** **(0–3)**	**Problem coping** **(1–4)**	**Emotion coping** **(1–4)**	**Avoidant coping** **(1–4)**
Mean (SD)	1.61 (1.10)	3.26 (1.10)	1.72 (0.52)	3.67 (0.89)	5.23 (1.16)	1.33 (0.60)	2.27 (0.60)	2.20 (0.37)	1.66 (0.41)
PTG	–	0.65^**^	−0.21^*^	0.24^**^	0.18^*^	0.27^**^	0.47^**^	0.25^**^	0.14
CBI		–	−0.18^*^	0.06	−0.04	0.10	0.45^**^	0.36^**^	0.36^**^
PA			–	−0.55^*^	−0.09	−0.06	−0.11	0.02	−0.06
PS				–	0.18^*^	0.18^*^	0.05	0.04	−0.01
SS					–	0.35^**^	0.12	0.08	−0.10
VS						–	0.17^*^	0.17^*^	−0.06
PC							–	0.46^**^	0.20^**^
EC								–	0.42^**^
AC									–

** p < 0.001;

* p < 0.01. For pet attachment only, lower scores indicate higher levels of attachment. PTG, Post-traumatic growth; CBI, Core belief disruption; Pet Attach, Pet attachment.

**Figure 1:**
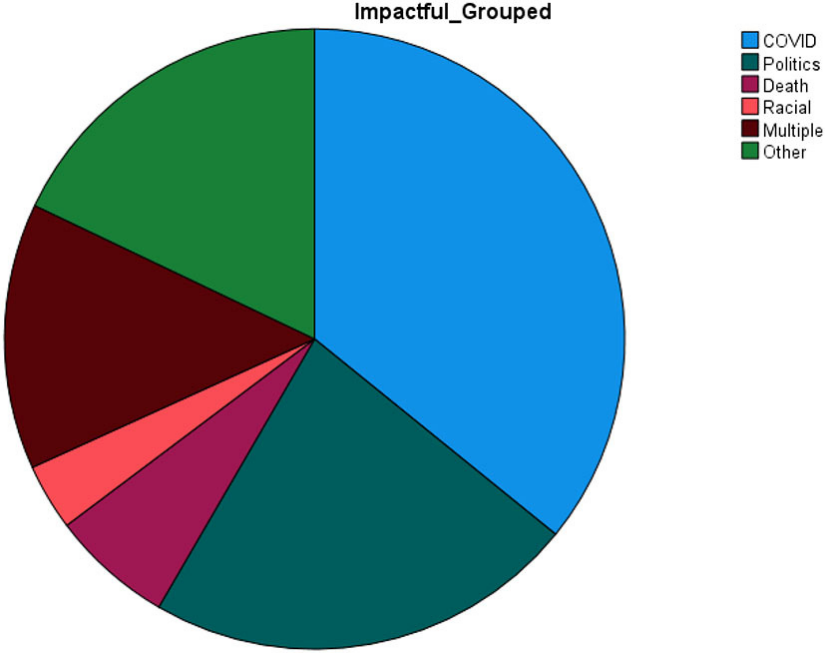
Most impactful event for participants by category.

Of the 201 participants, 28 did not indicate their most stressful event of the past year and were not included in the following percentages. Of the 173 remaining, 35.8% (*n* = 62) reported COVID-19 as the most stressful event of the past year, 22.5% (*n* = 39) rated politics as the most stressful event of the past year, 13.9% (*n* = 24) rated multiple events (i.e., COVID and politics) as the most stressful, and 17.9% (*n* = 31) rated “other events” as the most stressful. Please see Figure 1 for complete comparisons of the most impactful events.

### Group differences

The 201 participants who completed all four time points were significantly older [*t*_(270.94)_ = −5.46, *p* < 0.001; *M*_*complete*_ = 35.39, *M*_*non*−*complete*_ = 29.31; *d* = −.049] and reported slightly lower T4 PTG than those who completed three or less time points [*t*_(263)_ = 2.08, *p* = 0.038; *M*_*complete*_ = 1.60, *M*_*non*−*complete*_ = 1.91; *d* = 0.28]. Participants who owned pets reported higher total PTG than those who did not [*M*__*Pe*_t_ = 1.75, *SD* = 1.09; *M*_*NoPet*_ = 1.26, *SD* = 1.05; *t*_(183)_ = 2.85, *p* = 0.005; 95% CI: 0.139–0.773; *d* = 0.46], as did participants who knew someone who had died from COVID-19 [*M*_*Yes*_ = 1.79, *SD* = 1.07; *M*_*No*_ = 1.45, *SD* = 1.11; *t*_(182)_ = −2.08, *p* = 0.039; 95% CI: −0.602–−0.016; *d* = −0.31], and those who knew someone hospitalized from COVID-19 [*M*_*Yes*_ = 1.80, *SD* = 1.09; *M*_*No*_ = 1.36, *SD* = 1.08; *t*_(182)_ = −2.76, *p* = 0.006; 95% CI: −0.702–−0.115; *d* = −0.41]. There was no difference in total PTG between those who tested positive for COVID-19 over the year or those who knew someone who had tested positive and those who did not. Participants who rated COVID-19 as the most stressful event, those who had rated politics as the most stressful event, or those who gave multiple events as the most stressful also did not show a difference in total PTG, although those who considered the event resolved had significantly higher PTG than those who considered the event ongoing [*M*_*Closed*_ = 2.08, *SD* = 1.13, *M*_*Ongoing*_ = 1.49, *SD* = 1.07; *t*_(181)_ = 2.97, *p* = 0.002; 95% CI: 0.198–−0.981; *d* = 0.55]. There was also no difference in total PTG between those who lived alone and those that lived with others, people who had been vaccinated compared to the unvaccinated, high-risk individuals, essential workers, or those who lived with either a high-risk individual or essential worker compared to those who did not.

### Changes over time

From April 2020 until April 2021, core belief disruption significantly increased [*F*_(2.87, 499.38)_ = 15.96, *p* < 0.001; η^2^ = 0.084], as did growth in the new possibilities [*F*_(2.59, 455.23)_ = 15.37, *p* < 0.001; η^2^ = 0.080] and personal strength domains of PTG [*F*_(2.75, 488.85)_ = 3.34, *p* = 0.022; η^2^ = 0.018]. Total PTG and the domains of relating to others, spiritual change and appreciation of life did not significantly change over the year. Perceived support from pets decreased [*F*_(2.74, 273.51)_ = 15.26, *p* < 0.001; η^2^ = 0.132], as did use of video conferencing from May 2020 until April 2021 [*F*_(1.92, 347.08)_ = 17.05, *p* < 0.001; η^2^ = 0.086], but attachment to pets, use of touch with pets for support, and human social support remained stable over the year. Use of all coping strategies decreased [problem-focused coping: *F*_(3, 531)_ = 4.45, *p* = 0.004; η^2^ = 0.024; emotion-focused coping: *F*_(2.84, 482.02)_ = 2.91, *p*=0.035; η^2^ = 0.017 avoidant coping *F*_(2.78, 500.31)_ = 17.41, *p* < 0.001; η^2^ = 0.088]. Please see Table 3 for the mean ratings of PTG, social support variables, and coping strategies, across time points.

**Table 3:** Changes across time.

	**Range**	**Time 1**	**Time 2**	**Time 3**	**Time 4**
Total PTG	0–5	1.46 (0.96)	1.51 (1.02)	1.50 (1.11)	1.60 (1.10)
Relating to others	0-5	1.73 (1.15)	1.71 (1.21)	1.63 (1.30)	1.66 (1.19)
**Personal strength**	0–5	1.55 (1.23)	1.67 (1.29)	1.71 (1.42)	1.86 (1.46)
Appreciation of life	0–5	2.19 (1.33)	2.16 (1.38)	2.15 (1.36)	2.20 (1.46)
**New possibilities**	0–5	1.27 (1.01)	1.49 (1.10)	1.64 (1.30)	1.86 (1.34)
Spiritual change	0–5	0.81 (0.94)	0.83 (0.98)	0.78 (1.02)	0.85 (1.05)
**Core belief disruption**	1–6	2.80 (1.10)	2.93 (1.10)	3.19 (1.18)	3.25 (1.19)
Social support	1–7	5.37 (1.08)	5.26 (1.29)	5.25 (1.29)	5.23 (1.16)
**Video conferencing**	0.13–3	–	1.55 (0.66)	1.50 (0.63)	1.33 (0.60)
**Pet support**	1–5	4.05 (0.75)	3.77 (0.80)	3.57 (1.00)	3.67 (0.89)
Pet touch	0–4	–	3.04 (0.85)	3.00 (0.92)	3.09 (0.83)
Pet attachment	1–3.91	1.76 (0.52)	1.76 (0.51)	1.70 (0.46)	1.72 (0.51)
**Problem-focused coping**	1–4	2.40 (0.58)	2.27 (0.63)	2.29 (0.60)	2.27 (0.60)
**Emotion-focused coping**	1–3.83	2.27 (0.39)	2.23 (0.40)	2.28 (0.41)	2.20 (0.37)
**Avoidant coping**	1–3.5	1.87 (0.44)	1.83 (0.44)	1.79 (0.45)	1.66 (0.41)

Mean scores reported. Standard deviation in parentheses. Bold indicates significant change across time. Range indicates score range of data. For Pet Attachment, lower scores indicate higher levels of attachment. Video conferencing and Pet touch data was not collected at Time 1.

### Regression analyses

Hierarchical regression analyses revealed that core belief disruption had the largest impact on Time 4 PTG, resulting in significant overall models for all three regressions that accounted for a large amount of the variance in PTG. Two social support models were assessed, one on the full sample which accounted for 46% of the variance in PTG and one on pet owners only, which accounted for 56% of the variance. Along with core belief disruption, human social support was a significant predictor for both models, while support through video conferencing predicted PTG for the full sample only. None of the pet support variables significantly predicted PTG. The coping regression accounted for 45% of the variance in PTG and was predicted by both problem-focused coping and avoidant coping. See Table 4 for the complete social support regression results and Table 5 for the coping strategies regression results.

**Table 4:** Social support hierarchical regression results.

	**Full sample (*N* = 201)**	**Pet owners only (*n* = 142)**
Age	β = 0.01, *p* = 0.935 [−0.01, 0.01]	β = 0.03, *p* = 0.672 [−0.01, 0.02]
Sex	β = −0.02, *p* = 0.691 [−0.35, 0.23]	β = −0.10, *p* = 0.920 [−0.76, 0.12]
Household count	β = 0.07, *p* = 0.920 [−0.14, 0.15]	β = 0.01, *p* = 0.947 [−0.16, 0.17]
T4 CBI	***β*** **= 0.62**, ***p*** **< 0.001** **[0.46, 0.68]**	***β*** **= 0.73**, ***p*** **< 0.001** **[0.51, 0.75]**
Pet owner	β = −0.07, *p* = 0.232 [−0.46, 0.11]	–
Pet support	–	β = 0.10, *p* = 0.245 [−0.09, 0.34]
Pet touch	–	β = 0.15, *p* = 0.084 [−0.03, 0.46]
Pet attachment	–	β = 0.04, *p* = 0.631 [−0.32, 0.53]
T4 SS	***β*** **= 0.15**, ***p*** **= 0.021** **[0.02, 0.26]**	***β*** **= 0.22**, ***p*** **= 0.005** **[0.08, 0.40]**
T4 video support	***β*** **= 0.18**, ***p*** **= 0.026** **[0.04, 0.61]**	β = −0.06, *p* = 0.510 [−0.42, 0.21]
Video frequency	β = −0.05, *p* = 0.536 [−0.08, 0.04]	β = 0.11, *p* = 0.206 [−0.03, 0.12]
Model 1	***F*****_(4, 154)_ = 27.29**, ***p*** **< 0.001**	***F*****_(4, 94)_ = 24.28**, ***p*** **< 0.001**
Model 2	***F*****_(5, 153)_ = 22.60**, ***p*** **< 0.001**	***F*****_(7, 91)_ = 15.96**, ***p*** **< 0.001**
Model 3	***F*****_(8, 150)_ = 17.81**, ***p*** **< 0.001**	***F*****_(10, 88)_ = 13.71**, ***p*** **< 0.001**
Change 1 *R^2^* (Δ *R^2^, p*)	0.010, *p* = 0.105	**0.043**, ***p*** **= 0.039**
Change 2 *R^2^* (Δ *R^2^, p*)	**0.062**, ***p*** **< 0.001**	**0.058**, ***p*** **= 0.007**
% Variance	46.0%	56.5%

Model 1: sex, age, household count, CBI; Model 2 full sample: pet ownership; Model 2: pet owning sample: pet attachment, support from pets, pet touch; Model 3: human social support, video conferencing, video frequency. Significant predictors and models are bolded. [] = 95% Confidence Interval. % variance = Adjusted R^2^. CBI: Core belief disruption. Negative beta values indicate males are lower for sex and pet owners are higher than non-owners.

**Table 5:** Coping strategies hierarchical regression results.

	**T4 PTG**
Age	β = 0.03, *p* = 0.673 [−0.01, 0.01]
Sex	β = 0.01, *p* = 0.896 [−0.26, 0.30]
Household count	β = 0.02, *p* = 0.799 [−0.13, 0.16]
T4 CBI	***β*** **= 0.59**, ***p*** **< 0.001 [0.42, 0.67]**
T4 Problem-focused coping	***β*** **= 0.22**, ***p*** **= 0.002 [0.15, 0.67]**
T4 Emotion-focused coping	β = 0.01, *p* = 0.885 [−0.38, 0.44]
T4 avoidance coping	***β*** **= −0.14**, ***p*** **= 0.036 [−0.72,−0.03]**
Model 1	*F*_(3, 160)_ = 0.16, *p* = 0.923
Model 2	***F*****_(4, 159)_ = 29.52**, ***p*** **< 0.001**
Model 3	***F*****_(7, 156)_ = 20.70**, ***p*** **< 0.001**
Change 1 *R^2^* (Δ *R^2^, p*)	**0.423**, ***p*** **< 0.001**
Change 1 *R^2^* (Δ *R^2^, p*)	**0.055**, ***p*** **= 0.001**
% Variance	45.8%

Model 1: sex, age, household count; Model 2: CBI; Model 3: problem-focused coping, emotion-focused coping, avoidance coping. Significant predictors and models are bolded. [] = 95% Confidence Interval. % Variance = Adjusted R^2^. CBI: Core belief disruption.

## Discussion

The COVID-19 pandemic disrupted life as we knew it, but also brought about the opportunity to experience growth. This study examined changes in mental health, social support, and coping strategies in adults living in the United States over the first year of the pandemic and during the 2020 presidential election, from March 30th, 2020, until March 30th, 2021. Almost all participants (92.4%) knew someone who had contracted COVID-19 over that year and more than four out of ten knew someone who had died from the virus. More than one third of the sample rated COVID-19 as the most stressful event of the past year while another 22% rated politics as the most stressful. It was found that core beliefs about the world were disrupted during this year, consistent with previous studies on pandemics (Brooks et al., 2020), yet psychological growth was also experienced. The degree of psychological growth perceived was impacted by experiences with the pandemic, social support, and coping strategies, consistent with the PTG theoretical model (Calhoun and Tedeschi, 2006) and prior research on coping strategies (Hamama-Raz et al., 2019).

### Changes over time

During the year, participants experienced disruptions to their core schemas about the world, and the level of disruption to these core beliefs continued to increase throughout the year. This is consistent with prior research on quarantining which found reports of anxiety, stress, and post-traumatic stress symptoms that increased along with the duration of the quarantine (Brooks et al., 2020). While disillusionment with the world increased over the year, perceptions of overall PTG started at a moderately elevated level 2 weeks into the pandemic compared to other ratings of PTG during the early days of COVID-19 (Shigemoto, 2021) yet only showed incremental increases throughout the year. It is important to note that despite the moderately higher levels of PTG reported in this sample during early COVID-19, the rate of PTG found during COVID-19 is lower than rates of PTG found after more specific traumatic events, such as natural disasters (Cao et al., 2018).

Despite overall perceptions of growth remaining stable, participants did report significant increases of growth in the new possibilities and personal strength domains of PTG. These changes indicate that as the year progressed participants became more comfortable and confident with the “new normal” of life with COVID-19 and the changes that entails, including changes in work or hobbies. Indeed, although specific changes in habits were not assessed in this study, a survey conducted in early 2021 found that 59% of participants had taken on a new hobby during the pandemic and 79% of those reported wanting to continue their new hobby once the pandemic was over (Schulz, 2021). Additionally, a survey conducted in late 2020 found that 71% of workers were currently working from home, up from 20% before the coronavirus outbreak, and that 54% wanted to work from home after the pandemic ends (Parker et al., 2020), revealing substantial changes from the pre-pandemic work force. The steady increase of PTG in the personal strength domain demonstrates the increasing confidence that participants appeared to have in themselves and their ability to cope with the pandemic and other challenges they may have faced during that year. As time progressed and life went on, participants recognized more strength in themselves than they had previously realized they possessed.

### Factors impacting growth

#### COVID-19

Specific circumstances also appeared to impact the degree of psychological growth experienced by participants. Those who had more closely experienced the devastation of the pandemic through knowing someone who became ill enough to be hospitalized or who was killed from the virus experienced more growth than those who did not know someone seriously harmed by the virus. Intriguingly, participants who contracted the virus themselves or who had to be hospitalized because of COVID-19 did not show the same increase in PTG compared to those who did not get sick. This apparent paradox lends support to findings of a curvilinear relationship between stress or trauma and PTG found in adults (Kleim and Ehlers, 2009), where either too little or too much stress limits the degree of PTG experienced. Not knowing someone seriously impacted by the pandemic may correspond with lower overall levels of stress but becoming seriously ill yourself may result in stress levels that are too high to recognize growth. Alternatively, different trauma types have been proposed to have differing impacts on PTG, with personal traumas associated with less growth than shared traumas (Kilic et al., 2015). Becoming seriously ill from COVID-19 can be considered a personal trauma compared to knowing someone who became seriously ill, which may then correspond with the differing levels of growth seen in this sample.

#### Nature of stressor

While participants differed in which events during 2020 were the most stressful for them, these differences did not result in differences in PTG. As both political turmoil and waves of the pandemic were still ongoing in April of 2021 and both are shared traumas, it follows that there would not be significant differences in levels of PTG between those considering each event their most impactful stressor. Rather, perceptions of the event as either ongoing or resolved impacted levels of PTG, with those who thought of the event as resolved reporting significantly higher PTG than those who perceived the most stressful event to be ongoing. These findings support the PTG theoretical model, which emphasizes the importance of rumination and meaning making in the development of PTG (Calhoun and Tedeschi, 2006). If the stressful event is considered ongoing there may be less ability to reflect on its meaning and less ability to search for positive outcomes than if the event is considered resolved and in the past. Resources are more likely to be spent coping with the event if it is ongoing rather than processing the event, as is possible when the event is over.

#### Social support

Although social support was predictive of PTG in this study and this study occurred during a time when human social contact was more limited, whether a person lived alone or not or how many people they lived with did not have an impact on PTG. However, pet ownership did have an impact, with those who owned pets reporting higher levels of PTG than those who did not own pets. Attachment to pets and perceived support from pets were both moderately correlated with PTG; participants who were more attached or who perceived more support from their pets reported higher levels of PTG than those who were less attached or who perceived less support. Despite this, neither pet ownership nor any facet of pet support was predictive of experiencing PTG in the regression analyses. These contradictory findings may be explained by the several types of analyses—while pets and perceiving support from pets may provide a degree of social support that is helpful in the development of PTG when considered in isolation, support from pets may be less important and play a smaller role than support from humans when both types of social support are considered together. This would correspond with the prior research which found that more time spent with pets corresponded with higher growth in the relating to others domain of PTG for adolescents (Dominick et al., 2019), which assessed the impact of pets independently from the impact of human social support.

It is also possible that support from pets may have a larger role when support from humans is more limited. Indeed, a previous analysis of the first two time points from this study found that attachment to pets 2 weeks into the pandemic predicted PTG 1 month later when social distancing regulations were more enforced, and the vaccine was not available. However, attachment to pets was still a weaker predictor of PTG than was support from humans (Dominick et al., 2021). Additionally, perceived support from pets declined over the year, indicating that as restrictions on human contact lessoned, so too did reliance on pets for social support.

Taken together, it can be concluded that pet ownership and perceiving support from pets may have a small positive impact on perceptions of PTG, but that human support remains a more crucial factor for PTG compared to pets. Comparing the two social support regressions lends additional credence to this hypothesis. In the full sample, both human social support and perceived support through video conferencing were predictive of PTG, yet in the pet owners only sample, video support no longer had a significant impact on perceptions of PTG. Pets may supplement the support garnered from humans, rendering virtual support less important for pet owners than for non-pet owners. However, for those who do not have pets, virtual support, especially when in-person contact is more limited, appears to be an effective method to garner social support, consistent with prior research (Siniscarco et al., 2017; Viswanathan et al., 2020), and can contribute to the experience of PTG.

#### Core belief disruption

Along with social support and consistent with the PTG theoretical model, core belief disruption was predictive of experiencing PTG. The PTG theoretical model states that psychological growth is experienced after a person, with encouragement from social support, engages in rumination and meaning making, which are triggered not by the stressful event itself but by the impact of the event on a person's core beliefs about the world (Calhoun and Tedeschi, 2006). To that end, core belief disruption has consistently been found to predict PTG (Cann et al., 2010) and the relationship between core belief disruption and PTG has been found to be mediated by rumination (Taku et al., 2015). In this study, increased disruption of core beliefs corresponded with higher reported PTG, and core belief disruption was the driving force behind all three regression models. In fact, core belief disruption had a stronger impact on PTG than any measure of social support or coping strategy. However, combining social support measures with core belief disruption predicts approximately half of the variance in PTG observed in this sample, again providing additional support for the PTG theoretical model.

#### Coping strategies

Although core belief disruption had a larger impact than social support or coping strategies on PTG, coping strategies were predictive of PTG. Specifically, those who engaged in higher levels of problem-focused coping and lower levels of avoidance coping were more likely to experience PTG. Yet, participants appear to have decreased their use of coping strategies in general over the year. While emotion-focused coping was positively correlated to PTG, it was not a significant predictor when considered in tandem with problem-focused and avoidance coping strategies. However, this may be due to the lower reliability seen for the emotion-coping subscale in this sample. In general, avoidance coping is considered a maladaptive coping strategy while problem-focused coping is considered an adaptive coping style because each are associated with negative and positive mental-health outcomes, respectively, while emotion-focused coping can be considered as either adaptive or maladaptive depending on the situation (Carver, 1997). The results from this study replicate prior research which has found that both adaptive (problem-focused) and maladaptive (avoidance) coping were linked to PTG in samples of healthcare workers and 911 telecommunicators (London et al., 2017; Kalaitzaki and Rovithis, 2021). As healthcare workers and 911 telecommunicators are exposed to highly stressful or traumatic events over a prolonged period due to the nature of their jobs, perhaps these samples more accurately represent what many people in the United States were feeling during the year from April 2020 until April 2021, where the “stressful event” was not singular nor limited in time. Lending additional credence to this, lower levels of avoidance coping were associated with higher PTG for those who had experienced multiple traumas (Brooks et al., 2019) and for those in this study, who again may have perceived the year as a series of cumulative stressors rather than a single traumatic event. Thus, which coping strategies are the most effective may vary with the nature of the stressor—whether it is ongoing or sudden, how expected/unexpected it is, and whether it is a single event or cumulative events. In the case of the pandemic and the political turmoil in the United States during that year, increased use of problem-focused coping and decreased use of avoidance coping both appear to be effective at predicting the experience of PTG.

### Implications

Overall, this study lends additional credibility to the PTG theoretical model by highlighting the importance of disruptions to core assumptions, coping strategies, and social support to the development of psychological growth, as has been found in previous community studies (e.g., Gul and Karanci, 2017). Disruptions to core beliefs plays the largest role in predicting post-traumatic growth, however social support and the use of various coping strategies when rebuilding shattered world views also contribute to the perception of growth, accounting for almost half of the variance seen in PTG. While human social support is clearly the most effective source of social support in fostering growth, a sense of support may also be garnered from pets and through virtual communication. These alternative sources of support, while they do not compensate for in-person social support, may be valuable tools during times in which in-person contact is limited.

Given the elevated levels of stress and anxiety observed in our society currently (Bethune, 2020b; American Psychological Association, 2021), methods that can help increase positive psychological growth are needed. Based on this study, intervention programs should focus on assisting clients with processing their assumptive beliefs about the world and how those may have changed, increasing perceptions of social support, and teaching adaptive, problem-focused coping methods. While in-person social support should be encouraged, for those who are more isolated non-traditional forms of social support may also be valuable. These include video conferencing and virtual communication along with the comfort and support that may be provided through animals. Additionally, focusing on encouraging problem-focused coping and lessening the use of avoidance coping would be valuable for fostering psychological growth.

## Limitations and future directions

This study does have limitations. First, there was a high attrition rate over the course of the year, and it is possible that differences between those who choose to remain in the study and those who dropped out may have impacted the results. Those who completed the entire study were older than those who did not, likely due to university students who enrolled *via* the subject pool for research credit with the initial survey not responding to follow-up survey requests. The difference in PTG between groups may have resulted from additional stressors (such as from politics) that were present toward the second half of the study, which may have appeared more ongoing than the pandemic and may have impacted overall results. However, there were no other significant differences between non-completers and those who completed all four time points, so the impact on results should not be drastic. Second, the sample skews toward white, female, pet owners, which may limit generalizability. Similarly, most of this sample chose to get the vaccination when it became available. However, given the politicized nature of the vaccine, this may indicate a lack of diversity in the sample regarding political views. A more diverse and representative sample may have highlighted additional differences that were masked by the heterogeneity of this sample. Third, some measures were added as the study progressed, such as the impact of touch with pets on social support, limiting the possibility of assessing changes throughout the entire year. It is possible different patterns of change may have been observed had all variables been included from the beginning.

Although most social distancing policies have been lifted, future studies should continue to investigate the use of non-traditional sources of social support and their impact on mental health. Additionally, studies should continue to evaluate the use of various coping strategies after experiencing a variety of stressful events to help determine the most effective coping strategies for prompting psychological growth and whether they differ for shared vs. individual traumas and single vs. cumulative events.

Interventions aimed at fostering psychological growth should focus on the importance of social support and of problem-focused coping strategies, as well as assisting with examining and rebuilding core schemas about the world. Emphasis should be placed on the possibility of multiple forms of social support, such as virtual support and support through pets, depending on the unique situation of individuals. Those who can process through shaken beliefs using problem-focused coping, with the assistance of social support, are the most likely to experience high levels of psychological growth.

## Data availability statement

The raw data supporting the conclusions of this article will be made available by the authors, without undue reservation.

## Ethics statement

The studies involving human participants were reviewed and approved by Oakland University Institutional Review Board. The patients/participants provided their written informed consent to participate in this study.

## Author contributions

Study design, data collection, analyses, and writing were conducted by WD.

## Conflict of interest

The author declares that the research was conducted in the absence of any commercial or financial relationships that could be construed as a potential conflict of interest.

## Publisher's note

All claims expressed in this article are solely those of the authors and do not necessarily represent those of their affiliated organizations, or those of the publisher, the editors and the reviewers. Any product that may be evaluated in this article, or claim that may be made by its manufacturer, is not guaranteed or endorsed by the publisher.
